# Anatomical Pathology of Subtle Lisfranc Injury

**DOI:** 10.1038/s41598-019-51358-8

**Published:** 2019-10-16

**Authors:** Naoki Haraguchi, Koki Ota, Takuma Ozeki, Shingo Nishizaka

**Affiliations:** 1 Department of Orthopaedic Surgery, St. Marianna University Yokohama Seibu Hospital, 1197-1 Yasashicho, Asahi-ku, Yokohama, Kanagawa 241-0811 Japan; 2 Department of Orthopaedic Surgery, Tokyo Metropolitan Police Hospital 4-22-1 Nakano, Nakanoku, Tokyo, 164-8541 Japan

**Keywords:** Pathology, Fracture repair, Epidemiology

## Abstract

The extent and patterns of Lisfranc joint complex disruption in subtle Lisfranc injuries have not been well clarified. We reviewed the direct intraoperative findings for 87 patients, examined computed tomography images that had been obtained preoperatively for 73 of the patients, and classified the injuries according to the Kaar  *et al*. criteria as the transverse type (instability between the first cuneiform [C1] and the second metatarsal [M2] and between the second cuneiform [C2] and M2) or longitudinal type (instability between C1 and M2 and between C1 and C2). Our patients’ injuries were classified as follows: longitudinal type (38%), transverse type (30%), transverse type and first tarsometatarsal (TMT) joint injury (20%), longitudinal type plus transverse type (7%), longitudinal type and first TMT joint injury (3%), and longitudinal type, transverse type, and first TMT joint injury (2%). In 11 patients, the longitudinal injury extended into the naviculo-first cuneiform joint. In 41 (56%) of the 73 patients for whom CT images were obtained, 1 or more fractures (not counting small avulsion fragments between C1 and M2) were found. Orthopedic surgeons should be aware of the various injury patterns possible in cases of subtle Lisfranc injury.

## Introduction

Subtle Lisfranc injuries due to low-energy trauma result in midfoot instability, and misdiagnosis or inadequate treatment can lead to considerable long-term disability, including chronic instability, degenerative arthritis, flatfoot deformity, and long-standing pain^1–3^. These injuries are distinct from those due to high-energy trauma, such as that occurring in a motor-vehicle accident or fall from a height and accompanied by fracture and/or dislocation of bones and joints. The clinical and radiographic evidence of Lisfranc injury due to low-energy trauma is subtle, and a high index of suspicion is necessary to diagnose such injury properly.

Despite the observation of only minor radiographic changes, underlying soft tissue damage can be severe in some patients with subtle Lisfranc injury. The injury can exist as an isolated rupture of the Lisfranc ligament or injury of various components of the tarsometatarsal (TMT) joint complex^4–11^. The Lisfranc ligament originates from the lateral surface of the medial cuneiform and inserts obliquely into the base of the second metatarsal. This ligament is the strongest supporting structure of the TMT joint complex^12^ and has been considered the inevitable focal point of Lisfranc injuries. Currently, there is little information available on the actual pathological characteristics of subtle Lisfranc injuries. Lack of agreement between investigators regarding the best fixation method^4,5,10,11,13,14^ may be due to the absence of comprehensive pathoanatomy data pertaining to subtle Lisfranc injuries.

Although it is generally accepted that various patterns of injury involving the TMT joint complex, i.e., Lisfranc joint complex, can result, the various patterns of disruption have not been well documented. A sound knowledge of the anatomical pathology should help investigators conduct appropriate basic research and pave the way for improved operative techniques and outcomes. Thus, we conducted a retrospective study covering a large patient series to clarify the pathoanatomy of subtle Lisfranc injuries. Our aims were to identify the specific patterns of injury and the prevalence of each pattern and to determine the prevalence of fracture among patients with a subtle Lisfranc injury.

## Materials and Methods

The study group comprised 87 patients (62 men and 25 women who ranged in age from 12 to 87 years [mean ± SD, 34.0 ± 15.3 years]) who underwent treatment for a subtle Lisfranc injury between March 2006 and December 2016. All such injuries had been diagnosed according to the Faciszewski *et al*. criteria^15^. The original Faciszewski *et al*. criteria for diagnosis of a subtle injury of the Lisfranc joint were three-fold and based on plain radiography findings^15^: diastasis between the bases of the first and second metatarsals (M1 and M2, respectively) that measures 2–5 mm on the anteroposterior radiograph; no other injuries of the foot, including fracture or subluxation of the fourth or fifth metatarsal cuboid articulation, seen on the oblique radiograph; no subluxation of the base of M1 relative to the first cuneiform (C1). Subsequent to the study of Faciszewski *et al*., radiographic analyses showed that an increase of 2 mm or more in distance from C1 to the base of M2, as determined by a comparison of weight-bearing anteroposterior radiographs of both feet, corresponds to significant Lisfranc injury^16,17^. We applied this criterion in addition to the second and third Faciszewski *et al*. criteria when we diagnosed subtle Lisfranc injury in our study patients, so patients with any radiographically identifiable fracture or dislocation in the Lisfranc joint complex (with the exception of the fleck sign [signifying a small avulsion fragment between C1 and M2]) were not included in the study group. The injuries occurred during sports (n = 60, 69%), routine daily activity (n = 21, 24%), traffic accidents (n = 3, 3%), or work activity (n = 3, 3%). The left foot was affected in 66 (76%) cases and the right foot in 21 (24%). We identified the specific joints involved in the injury by direct intraoperative visualization.

Transverse, sagittal, and coronal computed tomography (CT) images had been obtained for 73 (84%) of the 87 patients. All fractures detected on CT images that had not been detected on plain radiographs were noted. These included fracture of the base of a metatarsal, fracture of the cuneiform, appearing as a faint line, and small avulsed fragment(s) or impacted fragment(s) around the joints.

All injuries were treated by open reduction and internal fixation with 1 or more screws or dorsal locking plate(s) via a longitudinal dorsal approach, centering the incision over the first and second TMT joints, extending from the cuneiforms to the proximal metatarsals. This incision was adequate for us to check the status of the first, second, and third TMT joints as well as that of the intercuneiform joint (C1–C2). If complete rupture of the dorsal intercuneiform ligament was observed, we extended the incision slightly, both proximally and medially, to check the status of the naviculo-first cuneiform joint. The fracture was not displaced in most patients in whom fracture was detected by CT, so a reduction maneuver was not needed in these patients. Intraoperatively, after identifying specific injured joints by detecting ruptured dorsal ligament(s), we gently inserted a small bone elevator into the joint to identify joint instability. We also applied manual stress (abduction, adduction, or plantar flexion) to the foot to detect any joint instability.

We classified the injuries broadly, according to the Kaar *et al*. criteria^7^, as the transverse type (instability between C1 and M2 and between the second cuneiform [C2] and M2) or the longitudinal type (instability between C1 and M2 and between C1 and C2). In a transverse type injury, a joint between the third cuneiform and the third metatarsal (M3) may be involved. These injury patterns were originally defined on the basis of a cadaveric study; however, we have recognized that these 2 transection patterns correspond well with actual injury patterns, so we used this system as a basic classification system for our study. Our study was approved by the Institutional Review Board of Tokyo Metropolitan Police Hospital. (Approval No. 17-A03). The committee confirmed that all methods were performed in accordance with relevant guidelines and regulations. Informed consent was obtained from all participants.

## Results

**Figure 1:**
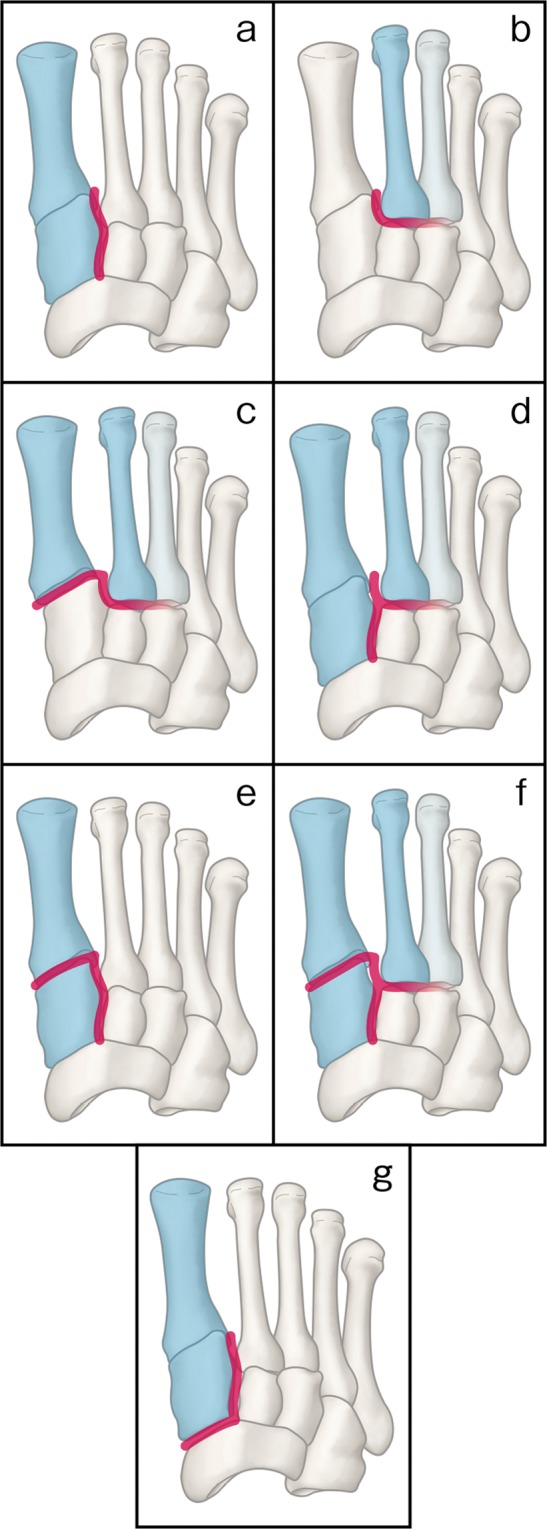
Various patterns of subtle Lisfranc injury. The injured joints are indicated in red. (**a**) Longitudinal type injury. (**b**) Transverse type injury. In some patients with this type of injury, the joint between the third cuneiform and third metatarsal may be involved. (**c**) Transverse type injury combined with first tarsometatarsal joint injury. (**d**) Longitudinal type injury plus transverse type injury. (**e**) Longitudinal type injury plus first tarsometatarsal joint injury. (**f**) Longitudinal type injury, transverse type injury, and first tarsometatarsal joint injury. (**g**) Longitudinal injury extending into the naviculo-first cuneiform joint.

**Figure 2:**
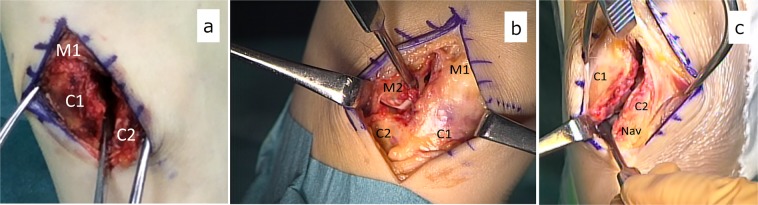
Intraoperative photographs depicting Lisfranc joint instability. The instability is confirmed with the use of a small elevator. (**a**) Longitudinal instability. (**b**) Transverse instability. (**c**) Longitudinal type injury extending into the naviculo-first cuneiform joint. C1: first cuneiform. C2: second cuneiform. M1: first metatarsal. M2: second metatarsal. Nav: navicular.

The patterns of injury detected are shown in Fig. 1. Our patients’ injuries were classified as follows: longitudinal type (n = 33, 38%) (Figs 1a and 2a), transverse type (n = 26, 30%) (Figs 1b and 2b), transverse type and first TMT joint injury (n = 17, 20%) (Fig. 1c), both longitudinal and transverse type injuries (n = 6, 7%) (Fig. 1d), longitudinal type and first TMT joint injury (n = 3, 3%) (Fig. 1e), and longitudinal type, transverse type, and first TMT joint injury (n = 2, 2%) (Fig. 1f). In 11 (13% of the total patients and 25% of the 44 patients with a longitudinal type injury), the longitudinal injury extended into the naviculo-first cuneiform joint (Figs 1g and 2c). A small avulsion fragment between C1 and M2 produced by avulsion of the M2 base or C1, appearing as a “fleck sign” on conventional radiographs, was observed in 24 (26%) of the 87 patients.

For analysis of injury mechanisms and injury patterns, we classified each injury as a relatively high-energy injury (sustained during sports activity or a traffic accident, n = 63) or a low-energy injury (sustained during routine daily activity or work activity, n = 24). We also classified each injury pattern as simple (simple longitudinal type injury without concomitant injury or simple transverse type without concomitant injury, n = 49) or complex (transverse type and first TMT joint injury; longitudinal type and first TMT joint injury; both longitudinal and transverse type injuries; combination longitudinal type, transverse type, and first TMT joint injury; or longitudinal type injury extending into the naviculo-first cuneiform joint, n = 38). Prevalence of the complex injury pattern did not differ significantly between patients with a high-energy injury (27 of 63 patients) and those with a low-energy injury (11 of 24 patients) (p = 0.80, chi-square test).

One or more fractures were detected on CT images of 57 (78%) of the 73 patients for whom CT images had been obtained, despite the fact that no fractures were detected on the radiographs of these 57 patients. A small avulsion fragment between C1 and M2 produced by avulsion of the M2 base or C1 was detected in 42 (58%) of the 73 patients. One or more fractures, including fracture of the cuneiform, appearing as a faint line and/or a metatarsal avulsion fracture around the TMT joint or the intercuneiform joint, or small impacted fragment(s) around the joints, but not counting small avulsion fractures of the M2 base or C1, were found in 41 (56%) of the 73 patients for whom CT images had been obtained.

Of the 41 patients in whom 1 or more fractures were detected by CT, 25 had a complex injury, whereas of the 32 patients without fracture, 6 had a complex injury, with the prevalence of complex injury being significantly greater among patients with fracture(s) (p = 0.003, by chi-square test).

## Discussion

Subtle Lisfranc injury often occurs in high-demand, physically active individuals who are involved in sports activities^1,2,8,16,18–20^. Persons recovering from this type of injury expect to have a normally functioning foot that is completely free of pain. The general consensus regarding treatment of Lisfranc joint injuries is that the key to a good outcome is stable anatomic reduction. However, there is no guarantee of a perfect outcome; such injury can become a source of persistent disability and pain, the amount of which varies from patient to patient^11,13^. Because orthopedic surgeons are becoming increasingly aware of the great potential for long-term disability resulting from subtle Lisfranc injury, foot injuries are being carefully screened and subtle Lisfranc injury is diagnosed with increasing frequency, particularly in sports participants^16,18^. Despite the growing number of reports on the treatment of subtle Lisfranc injury^2,8,13,15,17–19,21^, a relatively small number of patients have been studied, and the actual pathological characteristics of subtle Lisfranc injury remain unclear.

To the best of our knowledge, our subtle Lisfranc injury study group is the largest of such groups to be reported, and it allowed us to identify the specific injury patterns and prevalence of each pattern. A third of our study patients did not have a simple injury, i.e. transverse type or longitudinal type; rather, they had a combination of 2 or more injury patterns. Twenty-five percent of the total 82 patients had a concomitant first TMT joint injury, and 13% of the total patients (including 25% of the 44 patients with a longitudinal type injury) had a naviculo-first cuneiform joint injury. We also investigated whether fractures (not counting small avulsion fractures of M2 base or C1) had occurred and determined the prevalence to be 56%. The specific pathological characteristics of the injuries we identified are informative and will provide surgeons with a deeper understanding of the nature of Lisfranc instability.

The Lisfranc ligament is the strongest supportive structure of the TMT joint complex and has been considered to be the focal point of Lisfranc injuries. Some authors have suggested that subtle Lisfranc injury is caused by an isolated rupture of the Lisfranc ligament^21^. However, in a cadaver model, sectioning of the Lisfranc ligament alone was shown by Kaar *et al*. to be insufficient to produce radiographically identifiable instability of the Lisfranc joint^7^. They concluded that transverse instability required sectioning of both the Lisfranc ligament and the plantar ligament between C1 and M2 and M3 and that longitudinal instability required sectioning of both the Lisfranc ligament and the interosseous ligament between the C1 and C2^7^. Rupture of the Lisfranc ligament alone did not exist in any of our study patients with subtle Lisfranc injury, confirming the finding of Kaar *et al*.

Studies addressing Lisfranc injuries have failed to examine injury patterns in detail^2,8,18,21^, possibly because patients included in these studies underwent conservative treatment or percutaneous surgery. Nunley and Vertullo described stage II injuries as Myerson’s type B1 (partial medial incongruity) in 4 of their patients and as Myerson’s type B2 (partial lateral incongruity) in another^8^. Curtis *et al*.^2^ described 3 injuries (out of a total 19 TMT joint injuries) as type B1 injuries but failed to describe the injury patterns in the other cases. Deol *et al*.^19^ used a combination of the Nunley and Vertullo classification system^8^ and the Hardcastle *et al*. classification system^22^ to analyze and report outcomes in terms of return to training or competition. There were 7 Lisfranc ligamentous soft tissue disruptions classified as Nunley and Vertullo stage II (diastasis of 2–5 mm between M1 and M2 but no loss in arch height). The other 10 injuries had more significant bony malalignment and were graded according to the Hardcastle *et al*.^22^ system as 6 type B (partial incongruity) injuries and 4 type C (divergent, partial displacement) injuries. The Hardcastle *et al*.^22^ system, as modified by Myerson *et al*.^14^, is the most widely used classification system for injuries of the TMT joint complex. However, a shortcoming of these traditional classification systems is the lack of emphasis on the simple diastasis seen in low-energy athletic injuries. These classification systems deal mainly with fracture-dislocations of the Lisfranc joint complex and are not suitable for subtle Lisfranc injury.

Several experimental studies have been conducted in an effort to improve operative techniques. Using 14 cadaveric feet, Panchbhavi *et al*. compared the stability provided by a suture button with that provided by a screw when applied to diastasis associated with Lisfranc ligament injury^10^. They created a model by transecting only the Lisfranc ligament, leaving the dorsal intercuneiform ligaments and intermetatarsal ligaments intact^9,10^, and they concluded that suture-button fixation provides stability similar to that provided by screw fixation. In a cadaver study performed by Ahmed *et al*., who also compared suture button fixation against traditional interfragmentary screw fixation, surgical division of the dorsal and plantar Lisfranc ligaments was performed after dissection of superficial layers, and the intercuneiform ligaments were left intact^4^. They reported less displacement with standard interfragmentary screw fixation than with suture button fixation. Alberta *et al*. compared the ability of transarticular screws with dorsal plates to resist TMT joint displacement^5^. For this comparison, they created a ligamentous Lisfranc injury model by transecting the dorsal and plantar ligaments and capsule of all 5 TMT joints, as well as the dorsal, interosseous, and plantar Lisfranc ligaments. However, the intercuneiform and second through fifth intermetatarsal ligaments were left intact. In the study of Kadel *et al*.^6^, conducted to analyze the stabilizing function of the pointe shoe and Lisfranc ligaments in the ballet pointe position, a ligamentous Lisfranc injury model was created by sequential sectioning of the dorsal, interosseous, and plantar ligaments between the first metatarsocuneiform complex and second metatarsocuneiform complex as well as the first and second metatarsocuneiform joint capsule. Results of these previous studies, which compared different fixation methods for Lisfranc joint injury, should be interpreted with caution due to the limited representation of the spectrum of injuries. Because various injury patterns are possible, no single model of Lisfranc joint injury, especially a modest injury model, can represent the full spectrum of injuries, and thus generalizability of the study results is limited.

Percutaneous fixation is the most common treatment for subtle Lisfranc injury^20^. Because we found that subtle Lisfranc injury actually comprises a spectrum of injury patterns, we believe that open reduction is mandatory. It is very difficult to determine which joint is injured when a percutaneous procedure is performed, and some joints that are unstable joint may be overlooked. In our study, 22 patients (25% of the total patients) had a concomitant first TMT joint injury, and 11 patients (13% of the total patients and 25% of the 44 patients with a longitudinal type injury) had a naviculo-first cuneiform joint injury. For some concomitantly injured joints, the instability is slight, and these joints become stable after fixation of the adjacent unstable joint. However, according to our experience, most concomitantly injured joints tend to remain unstable even after fixation of the articulation between the base of M2 and C1, and additional fixation of these joints is often needed. These concomitant injuries can lead to chronic osteoarthritis if not surgically fixed. Our findings should alert orthopedic surgeons to the possibility of such concomitant injuries. The various injury patterns themselves can be used to determine the appropriate sites of fixation. Preoperative magnetic resonance imaging can be used to detect injured joints, and the images may serve as a useful intraoperative reference, though such imaging is of limited value for assessment of the degree of instability.

**Figure 3:**
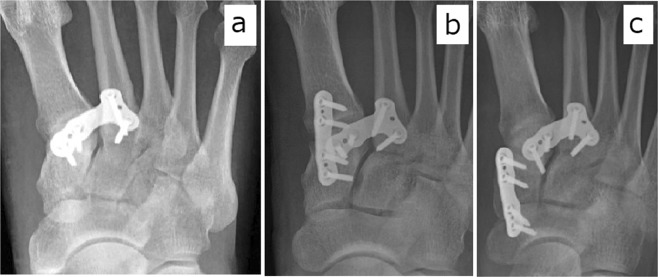
Postoperative radiographs of longitudinal type injury (**a**), transverse type injury combined with first tarsometatarsal joint injury (**b**), and Longitudinal injury extending into the naviculo-first cuneiform joint (**c**).

Our surgical decision-making process lies beyond the scope of our study. We note, however, that we have learned, over the study period, to observe some basic guidelines for Lisfranc injury fixation, as follows: In cases of simple longitudinal type injury, transverse type injury, or combination longitudinal and transverse type injury, we fix the joint between C1 and M2 (Figs 1a,b,d and 3a). In cases of a transverse type injury, the second TMT joint usually stabilizes after this C1–M2 fixation. In cases of a transverse type and first TMT joint injury (Fig. 1c), longitudinal type and first TMT joint injury (Fig. 1e), or combination longitudinal type, transverse type, and first TMT joint injury (Fig. 1f), we fix the first TMT joint as well as the joint between C1 and M2 (Fig. 3b). In cases of a longitudinal injury extending into the naviculo-first cuneiform joint (Fig. 1g), we fix the joint between C1 and M2 as well as the naviculo-first cuneiform joint (Fig. 3c). Additional study is needed to validate these guidelines.

The prevalence of fracture (56%) in our patients with subtle Lisfranc joint injury was greater than is generally believed, and there are diagnostic implications. That is, surgeons should be cautious not to miss a concomitant Lisfranc ligament rupture when a nondisplaced fracture is identified on a CT image. There is a tendency to diagnose this type of injury simply as a nondisplaced midfoot fracture and then to apply conservative treatment, which may lead to late diastasis between the base of the M2 and the C1. Furthermore, we found that the prevalence of complex injury patterns was significantly greater among patients with fracture(s) detected by CT than among patients without fracture(s). Therefore, we may be able to predict that if we detect 1 or more fractures on CT images (excluding small avulsion fractures of M2 base or C1), the injury pattern will be more complex, and we may need to check the status of the adjacent joint thoroughly for the existence of a complex injury, which generally needs additional fixation(s).

Results of our study should be interpreted in light of the study limitations. The major limitation is that study was retrospective; however, all data were collected prospectively. Because of the relative rarity of subtle Lisfranc joint injury, it is questionable whether a prospective study that includes a large patient series is feasible. Such a study would extend over an undesirably long period of time. Another limitation is that we did not confirm plantar ligament disruption directly. In addition, it might be argued that some of the injuries in our patients were too severe to be considered subtle. However, we applied the Faciszewski *et al*. criteria^15^ strictly, and all injuries fell into the subtle Lisfranc injury class. Lastly, CT was not performed for all patients. Early in the period covered by the study, CT studies were performed to assess spatial widening in some patients, and 1or more fractures were detected in some of these patients. From that time onward, we obtained CT images for all patients presenting with what was considered a subtle Lisfranc injury. The CT was performed to screen for fracture and, when necessary, to assess the C1–M2 distance.

In conclusion, subtle Lisfranc injury actually comprises a spectrum of midfoot joint injuries. Many of the associated injuries are being missed and conservative treatment or percutaneous surgery may not therefore be the correct approach. For patients with 1 or more fractures depicted on CT images (excluding small avulsion fractures of the M2 base or C1), a complex injury requiring additional fixation(s) can be expected. We believe the specific pathological characteristics of the injuries we identified will provide surgeons with a deeper understanding of the nature of the instability and may lead to more accurate and reliable reduction and fixation methods.
